# Auxiliary diagnosis of Lung Cancer on the basis of a Serum Protein Biomarker Panel

**DOI:** 10.7150/jca.57429

**Published:** 2021-03-15

**Authors:** Qiong Lu, Zhongwei Jia, Junli Gao, Meijuan Zheng, Junshun Gao, Mingjie Tong, Jinxing Xia, Fang Li, Baoling Yang, Lili Zhang, Bo Wang, Rui Wang, Jinping Qiao, Qinqin Lou, Jinbo Gao, Yuanhong Xu

**Affiliations:** 1 Department of Clinical Laboratory, the First Affiliated Hospital of Anhui Medical University, Hefei, 230031, China.; 2 Cosmos Wisdom Mass Spectrometry Center of Zhejiang University Medical School, Hangzhou, 311200, China.

**Keywords:** lung cancer, protein biomarker, nodule, cancer diagnosis, Chinese populations

## Abstract

**Objectives:** In this study, we established a serum protein biomarker panel (consisting of Pro-SFTPB, CA125, Cyfra21-1, and CEA) and evaluated the feasibility and performance for the auxiliary diagnosis of lung cancer in the Chinese population.

**Materials and Methods**: The current study was a single-center study based on the Chinese population and performed in two cohorts (training cohort and validation cohort). Serum concentrations of Pro-SFTPB, CA125, Cyfra21-1, and CEA were measured by a bead-based flow fluorescence immunoassay. The discrimination performance of the model was assessed using sensitivity, specificity, and the area under the receiver operating characteristic (ROC) curve (AUC).

**Results**: For the biomarker panel model, the AUC was 0.88 (95% CI, 0.85-0.91) in the training cohort and 0.90 (95% CI, 0.86-0.92) in the validation data cohort, which was significantly greater than the AUC of each biomarker alone. For the nodule risk model, the AUC was improved to 0.96 (95% CI, 0.94-0.98) in the training cohort and 0.95 (95% CI, 0.93-0.97) in the validation cohort. In addition, the biomarker panel model yielded an AUC of 0.78 (95% CI, 0.74-0.81) for stage I & II lung cancer, better than the performance of individual biomarker alone.

**Conclusions**: It was demonstrated that 4-protein biomarker panel had a significant performance in identifying lung cancer patients from healthy controls, especially combining with the nodule size. Specifically, it yielded excellent discrimination for identifying early-stage lung cancer patients than individual biomarker alone. A future large-scale study is underway to further define the clinical application of this method for the early diagnosis of lung cancer among Chinese populations.

## Introduction

Lung cancer is one of the most malignant forms of human cancers, and one of the leading causes of cancer mortality worldwide 1, 2. Lung cancer is highly correlated with tobacco exposure 3, with 2.1 million new lung cancer cases and 1.8 million deaths among smokers annually 1. Besides, its incidence among non-smokers remains a significant global health problem, especially in East Asia and most predominantly among women patients 3. In 2015, it was estimated that there were 610,200 lung cancer-associated deaths in China 4. Among them, more than 25% of the population were female lung cancer patients. The high lung cancer incidence rate among Chinese women, despite their low smoking prevalence, was thought to reflect increased exposures to environmental hazards, such as smoke from burning of charcoal for heating and cooking 3. Thus, developing effective methods of early lung cancer detections among both smoking and non-smoking populations remains a critical public health task, not only in China but also in the world.

Also in China, approximately 88.2% of the lung cancer patients are asymptomatic at initial and routine annual health screening. On the other hand, once diagnosed, approximately 65.3% of the lung cancer patients were already at stage III or stage IV 5. Several techniques, including thoracic radiography 6, sputum cytology 7, and computed tomography (CT) 8 are commonly adapted as auxiliary tools for lung cancer diagnosis. While thoracic radiography and sputum cytology have failed to provide adequate levels of sensitivity for early-stage lung cancer diagnosis, CT screening is often considered as a better imaging-based detection method and is recommended for lung cancer diagnosis among heavy smokers by the US Preventive Services Task Force (USPSTF) 9. On the other hand, the limitations of CT scanning are also well documented, including a high false-positive rate and inability to distinguish benighted nodules versus tumorigenic nodules 10. Thus, other auxiliary detection methods, such as biological and genetic screenings are much needed to efficiently identify lung cancer patients at early stages.

Among many of the auxiliary lung cancer detection methods, protein-based biomarker diagnostics seemed to be promising for early-stage cancer identification 11. During the past decades, protein-based biomarker investigations have been conducted in serum, tissue, and sputum, with serum being the least invasive and hence, most desirable testing matrix 12. Several serum biomarkers, such as carcinoembryonic antigen (CEA) 13, cytokeratin 19 fragment (Cyfra21-1) 14, tissue polypeptide antigen (TPA) 15, squamous cell carcinoma antigen (SCC) 16, stem cell factor (SCF) 17, granulocyte-macrophage colony-stimulating factor (GM-CSF) 18, and vascular endothelial growth factor (VEGF) 19 had been identified to be specifically associated with lung cancer. However, none of these protein biomarkers has achieved the desired sensitivity and specificity to be warranted as an independent diagnostic prospect. In that regard, a number of multianalyte panels comprised of both circulating proteins and tumor-associated autoantibodies have been developed and yielded encouraging results 20, 21. Unfortunately, most of these panels are still in pre-clinical stages and commercial products are not available.

In the current work, our single‐center study of a lung cancer biomarker panel found that 4 biomarkers in combination, including pro-surfactant protein B (Pro-SFTPB) 22, 23, carbohydrate antigen 125 (CA125) 11, Cyfra21-1 14, and CEA 13, can dramatically improve the accuracy and sensitivity of lung cancer diagnosis. Specifically, this panel exhibits superior performance for early-stage lung cancer detection than individual biomarker alone in both the training and validation cohorts. Notably, in combination with the nodule size, the detection performance was significantly improved. We hope the results of our study would serve as a promoting factor to facilitate a large-scale multiple-center clinical trial to further characterize the clinical feasibility of using this 4-protein biomarker panel for early diagnosis of lung cancer in China.

## Materials and methods

### Study design

The current study was aimed to evaluate the feasibility and performance of a combination of 4-protein biomarker panel (Pro-SFTPB, CEA, CA125, and CYRFRA21‐1) for the lung cancer diagnosis. Two models were established: in one, patients were assessed by the 4-biomarker panel model (biomarker panel model); in the other, patients were assessed by the model combined 4-biomarker panel with the nodule size (nodule risk model). Subsequently, scores derived from the logistic regression analysis in the training cohort were validated in a validation cohort.

### Blood samples

Blood samples were collected separately from two branches at the First Affiliated Hospital of Anhui Medical University. For the training cohort, a total of 180 patients with lung cancer and 360 matched controls were enrolled from October 2019 to June 2020. For the validation cohort, a total of 135 patients with lung cancer and 289 healthy controls were enrolled from November 2019 to July 2020. Patients were included if the following criteria were met: (a) no family history of lung cancer or personal history of malignant cancer; (b) not received chemotherapy or radiotherapy; and (c) no currently known extrathoracic malignant diseases. Upon retrieval, serum samples were immediately separated and stored at ‐80 °C, before further processing. All clinical data, including age, sex, medical history, pathological diagnosis, and imaging findings were collected and independently entered into a secured database. This study conformed to the ethical guidelines of the Declaration of Helsinki and was approved by the Medical Ethics Committee of the First Affiliated Hospital of Anhui Medical University (No. Quick-PJ 2020-13-13).

### Serum concentration analysis

**Figure 1:**
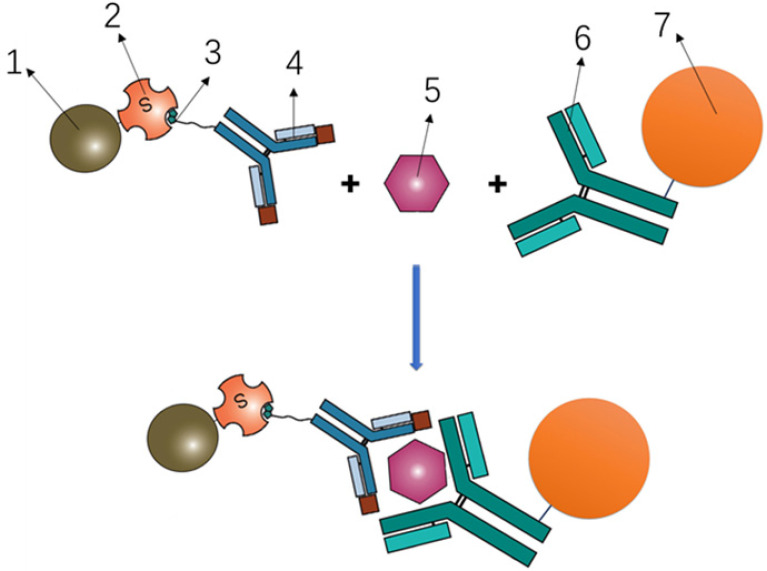
Principle of Luminex flow fluorescence capture sandwich immunoassay. 1- Phycoerythrin; 2- Streptavidin; 3- Biotin; 4- Labeled antibody; 5- Target protein; 6- Coating antibody; 7- Magnetic beads.

To determine the serum concentrations of Pro-SFTPB, CA125, CEA, and Cyfra 21-1, a bead-based Luminex flow fluorescence capture sandwich immunoassay was performed on the MAGPIX® platform (Luminex Corporation, Austin TX). The principle was shown in **Fig. 1**. Briefly, 2000 coupled beads were incubated with 20 μl of a serum sample for 1 hour, washed, and incubated with 50 μl of the detection antibody (4 μg/ml) for 30 minutes. The beads were washed again and incubated with 50 μl streptavidin-phycoerythrin (SA-PE, 4 μg/ml) for 15 minutes, followed by suspension in 125 μl of assay buffer and examination on the MAGPIX® instrument according to manufacturer's protocol. All experiments were carried out in duplicates, at room temperature, and protected from light. Calibration curves were established using 8 calibrators in a 2-fold dilution series. The coefficients of variation (CVs) within and between replicates were, 4.55% and 7.62% for Pro-SFTPB, 5.68% and 8.54% for CA125, 3.54% and 8.32% for CEA, 5.62% and 8.81% for Cyfra 21-1, respectively.

### Nodule assessment

The low-dosage chest CT examination was performed using the multiple contiguous sequential axial imaging procedure through the thorax. The nodule size was analyzed with the PneuView system (Myrian, Paris, France). The maximum dimension on axial CT images was recorded. Histological diagnosis was performed by at least two independent histologists. The clinical staging was determined according to the criteria of the 2004 World Health Organization classification of lung tumors 24, and the TNM staging criteria in the seventh edition of the American Joint Committee on Cancer staging manual 25.

### Statistical analysis

In this study, the averaged data were presented as mean ± the standard deviation (SD). Two predictive models, the biomarker panel model, and nodule risk model were initially developed within the training cohort. Then, their predictive performances, including discrimination (ability to classify) and calibration (ability to compare between predicted and observed probabilities) were further evaluated using the validation cohort. Model discrimination was performed using the receiver operating characteristic (ROC) analysis for lung cancer risk, including area under the ROC curve (AUC), sensitivity, and specificity. Statistical analysis was performed using the unpaired two-tail Student* t*-test (for age, nodule size, and serum level) and chi-square test (for sex and smoking status) on the MedCalc software (Version 19.4.1, MedCalc, USA) and the SPSS statistical software (version 22.0, IBM, USA). *P*< 0.05 was considered statistically significant.

## Results

### Subject characteristics

In the training cohort, a total of 180 patients with lung cancer (case group) and 360 controls (control group) were enrolled, including 112 non-smokers in the cases group and 233 non-smokers in the control group. The number of cases identified at TNM stage I& II and stage III & IV were 83 and 85, respectively. No significant difference was found in age and sex between the case and control group. The mean (±SD) nodule size was significantly larger for cases (2.3±2.0 cm) than that of controls (0.4±0.2 cm) (*P*<0.001). Compared with the cases, the smoking status was significantly different in the controls (*P*<0.001). The detailed subject characteristics for the training cohort are listed in **Table 1**.

In the validation cohort, a total of 135 patients with lung cancer and 289 controls were enrolled, including 83 non-smokers in the cases group and 195 non-smokers in the control group. In the case group, there were 55 subjects at early stage (I&II) and 69 subjects at advanced stage (III/IV). No significant difference was found in sex between the case and control group, while the mean (±SD) age of cases was older than that of controls (62.2±10.8 vs. 58.0±7.7, *P*<0.001). From November 2019 to July 2020, there were not enough older subjects enrolled in the control group. Besides, there was a significant difference in terms of nodule size and smoking status between the case and control group (*P*<0.001). The detailed subject characteristics for the validation cohort are listed in **Table 1**.

### Serum biomarker levels

**Figure 2:**
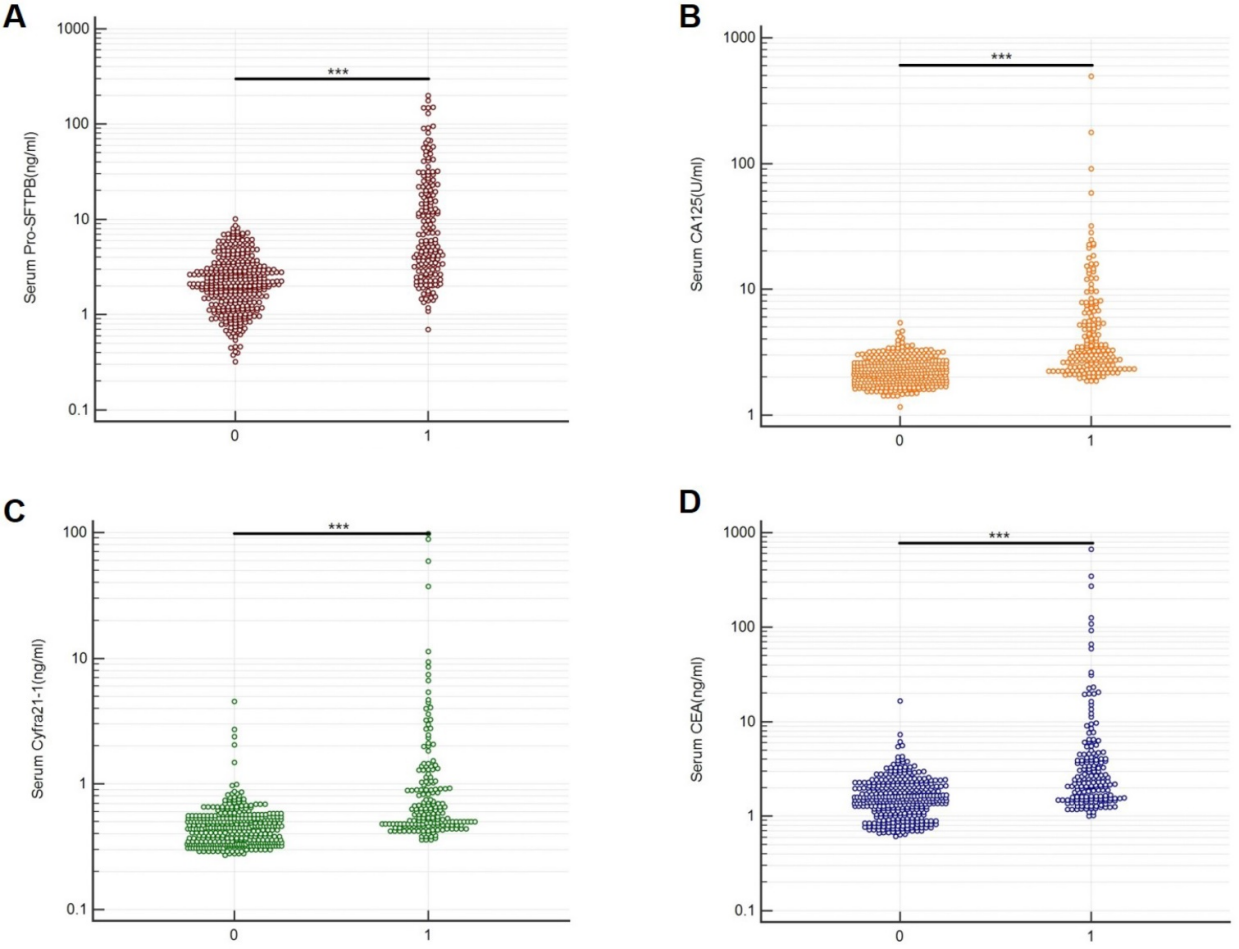
Serum biomarker levels in the training cohort. Serum protein concentrations of Pro-SFTPB (A), CA125 (B), Cyfra21-1(C), and CEA (D) were compared between the control group (0) and case group (1). ***P<0.001 vs. control by unpaired two-tail Student t-test.

A bead-based Luminex flow fluorescence capture sandwich immunoassay was used to detect the serum protein concentrations of Pro-SFTPB, CA125, Cyfra21‐1, and CEA in the training cohort and validation cohort. And the detection results were shown in **Table 2**. It was demonstrated that, in the training cohort, the concentrations of Pro-SFTPB, CA125, Cyfra21‐1, and CEA were significantly upregulated in the case group than in the control group (**Fig. 2**, *P*<0.001). In lung cancer patients, no correlation was found between 4 biomarker levels and age, sex (data not shown), smoking status, or nodule size (**Table 2**). Furthermore, serum levels of the 4 protein biomarkers in advanced stage (stage III&IV) cases were markedly higher than those in the early stage (stage I‐II) (**Table 2,** *P*<0.001). The level of Pro-SFTPB, CA125, and Cyfra21-1 were higher in patients with squamous cell carcinoma (SCC) than those with adenocarcinoma (ADC) and small cell cancers (SCLC). On the contrary, a lower level of CEA was found in patients with SCC (**Table 2**). Similar results were observed in the validation cohort (**Table 2**).

### Diagnosis performance of biomarker panel model

**Figure 3:**
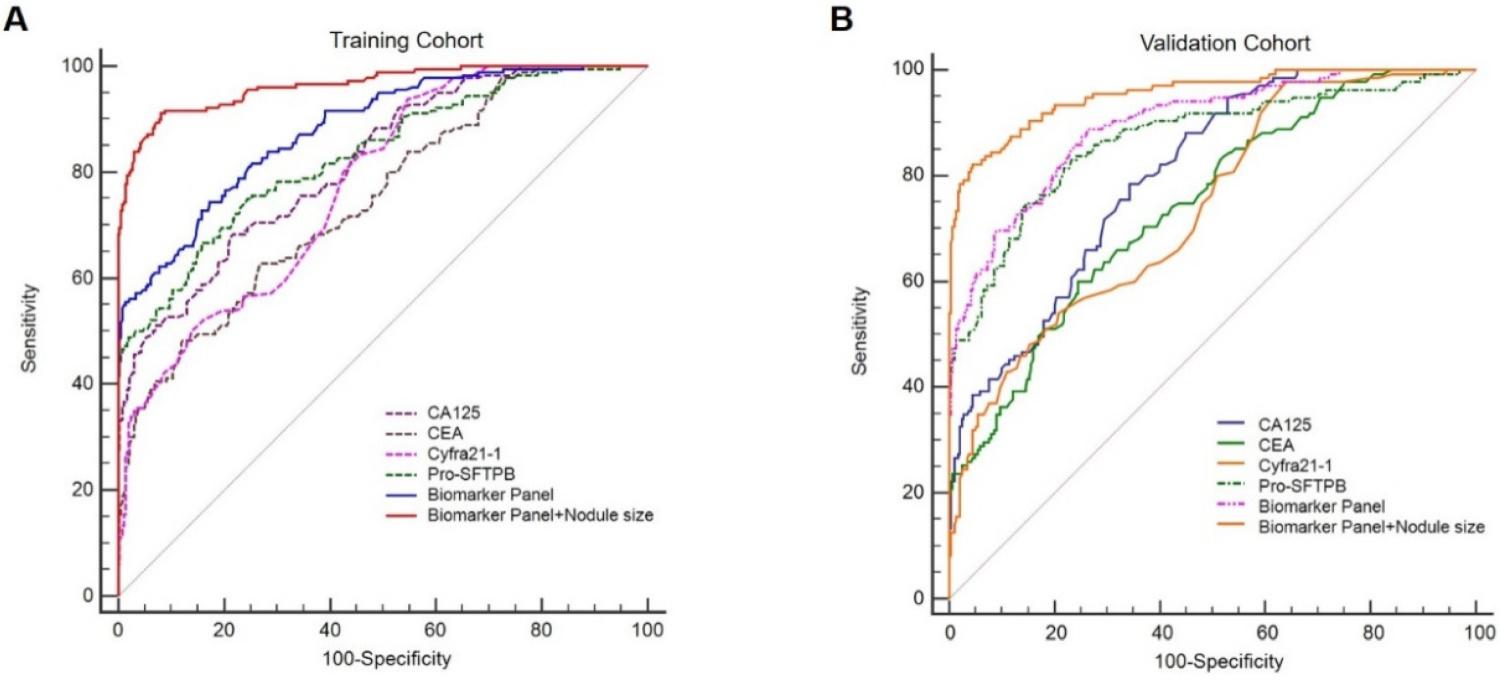
Receiver operating characteristic curves (ROCs) of Pro-SFTPB, CA125, Cyfra21-1, CEA, biomarker panel, and nodule risk model (biomarker panel + nodule size) for all lung cancer patients in the training cohort (A) & validation cohort (B).

Serum levels of 4 biomarkers (Pro-SFTPB, CA125, Cyfra21‐1, and CEA) were combined and produced a score by the logistic regression algorithm [**Table 3**, the logistic regression equation was logit (*P*) = -3.69+1.45×ln (Pro-SFTPB) +2.28×ln (CA125) +1.38×ln (Cyfra21-1)-0.32×ln (CEA)]. In addition, age, sex, smoking status, and medical history were not significant variables in the logistic regression (data not shown). The sensitivity and specificity for the 4-protein biomarker panel were calculated. In the training cohort, the sensitivity of the 4-protein biomarker panel was 57.8% at 95% specificity and 54.8% at 99% specificity. Similarly, the sensitivity of the 4-biomarker panel was 60.7% at the 95% specificity and 47.4% at the 99% specificity in the validation cohort (**Table 4**). Furthermore, the performance of individual biomarker, as well as in combination, in discerning lung cancer patients from healthy controls was assessed by the ROC analysis. It was demonstrated that the diagnostic performance of the biomarker panel was AUC=0.88 (95% CI, 0.85-0.91), significantly greater than that of each biomarker alone [**Fig. 3A,** biomarker panel (AUC=0.88) vs. Pro-SFTPB (AUC=0.83), *P*<0.0001; biomarker panel (AUC=0.88) vs. CA125 (AUC=0.82), *P*<0.0001; biomarker panel (AUC=0.88) vs. Cyfra21-1 (AUC=0.78), *P*<0.0001; biomarker panel (AUC=0.88) vs. CEA (AUC=0.75), *P*<0.0001]. This finding was also confirmed in the validation cohort, which showed excellent discrimination of biomarker panel, with AUC=0.90 (95% CI, 0.863-0.923) [**Fig. 3B,** biomarker panel (AUC=0.90) vs. Pro-SFTPB (AUC=0.87),* P*<0.05; biomarker panel (AUC=0.90) vs. CA125 (AUC=0.80), *P*<0.0001; biomarker panel (AUC=0.90) vs. Cyfra21-1(AUC=0.74), *P*<0.0001; biomarker panel (AUC=0.90) vs. CEA (AUC=0.74),* P*<0.0001].

**Figure 4:**
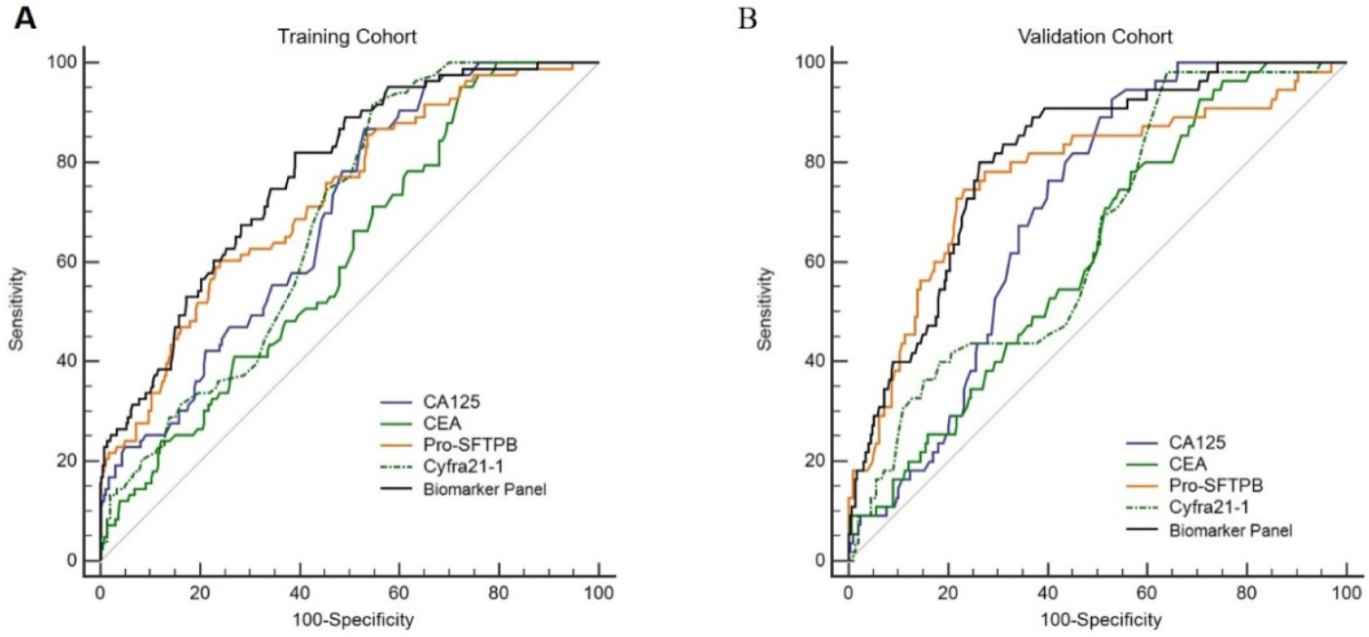
Receiver operating characteristic curves (ROCs) of Pro-SFTPB, CA125, Cyfra21-1, CEA, and biomarker panel in patients with stage I & II lung cancer in the training cohort (A) & validation cohort (B).

Specifically, the performance of the individual biomarker and biomarker panel was further assessed in subgroups. No matter biomarker panel or each biomarker alone, the diagnostic performance for the advanced stage lung cancer was effective (AUC>0.87 in the training cohort and AUC>0.80 in the validation cohort). To assess the diagnosis ability of the biomarker panel on early-stage of lung cancers, we focused on the patients in the training cohort with stage I & II disease. It was noticed that the diagnostic performance of the biomarker panel for early-stage lung cancer was better than that of each biomarker alone [**Fig. 4A,** biomarker panel (AUC=0.78) vs. Pro-SFTPB (AUC=0.73), *P*<0.0001; biomarker panel (AUC=0.78) vs. CA125 (AUC=0.70), *P*<0.001; biomarker panel (AUC=0.78) vs. Cyfra21-1 (AUC=0.68), *P*<0.001; biomarker panel (AUC=0.78) vs. CEA (AUC=0.61), *P*<0.0001]. This finding was then validated in the validation cohort, as the biomarker panel yielded a better result with an AUC=0.81 (95% CI, 0.76-0.85) for predicting early-stage lung cancer, better than CA125, CEA or Cyfra21-1 alone [**Fig. 4B,** biomarker panel (AUC=0.81) vs. CA125 (AUC=0.70), *P*<0.001; biomarker panel (AUC=0.81) vs. Cyfra21-1 (AUC=0.65), *P*<0.001; biomarker panel (AUC=0.81) vs. CEA (AUC=0.62), *P*<0.0001]. Interestingly, the diagnostic performance of Pro-SFTPB for early-stage lung cancer was significantly higher than the other three individual biomarker and comparable with that of the biomarker panel [**Fig. 4B,** Pro-SFTPB (AUC=0.77) vs. 4-biomarker panel (AUC=0.81), *P*=0.18], suggesting a potential independent predictor of Pro-SFTPB for early-stage lung cancer detection.

**Figure 5:**
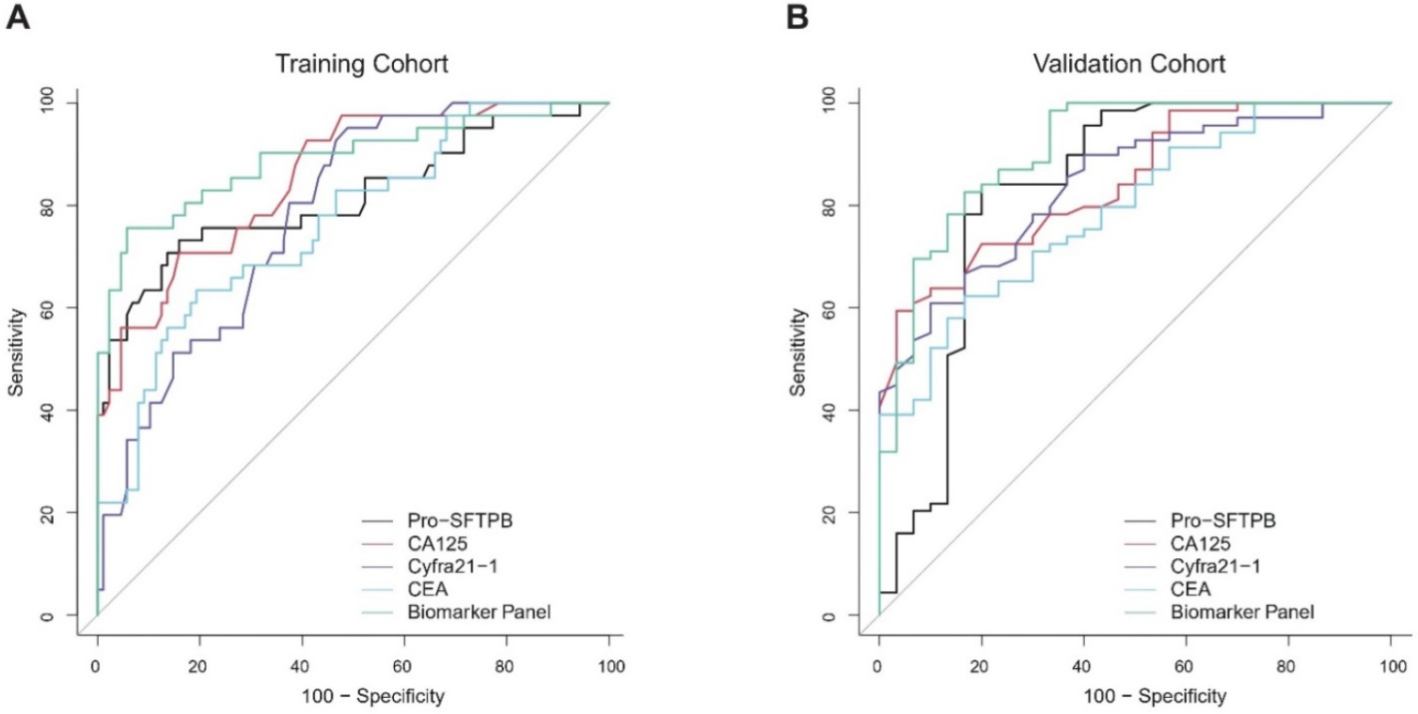
Receiver operating characteristic curves (ROCs) of Pro-SFTPB, CA125, Cyfra21-1, CEA, and biomarker panel in patients with small nodules (maximum diameter of nodule <1cm) in the training cohort (A) & validation cohort (B).

Moreover, there were a similar AUC magnitude between smokers and nonsmokers in both training and validation cohorts, owing to a lower proportion of smokers in the lung cancer patients (data not shown). Notably, we also assessed the diagnosis performance of the individual biomarker and biomarker panel for the small malignant nodules (maximum diameter of nodule <1 cm). As shown in **Fig. 5**, the biomarker panel model yielded an AUC of 0.89 (95% CI, 0.82-0.96) with a 70.7% sensitivity at 95% specificity in the training cohort (**Fig. 5A**), as well as an AUC of 0.91 (95% CI, 0.86-0.97) with a 65.7% sensitivity at 95% specificity in the validation cohort (**Fig. 5B**), showing the potential candidate markers for distinguishing the small malignant pulmonary nodules from normal individuals.

### Diagnosis performance of nodule risk model

The nodule risk model was developed by combining the biomarker panel with the nodule size. Logistic regression algorithm was performed to assess the detection performance of the nodule risk model [**Table 5,** logistic regression equation was logit (*P*) = -10.10+1.12×ln (Pro-SFTPB) +3.60×ln (CA125)+0.036×ln (Cyfra21-1)+0.48×ln (CEA) +7.63×(Nodule diameter)]. In the training cohort, the sensitivity of the nodule risk model for detecting lung cancer was 86.4% at the 95% specificity and 73.9% at the 99% specificity, which was validated in the validation cohort (**Table 4**). As compared with the biomarker panel model, the nodule risk model had significantly higher diagnostic accuracy for distinguishing lung cancer from normal individuals in either the training cohort [**Fig. 3A**, AUC of 0.96 (95% CI, 0.94-0.98) in the nodule risk model vs. AUC of 0.88 (95% CI, 0.85-0.91) in the biomarker panel, *P*<0.001] or the validation cohort [**Fig. 3B**, AUC of 0.95 (95% CI, 0.93-0.97) in the nodule risk model vs. AUC of 0.90 (95% CI, 0.86-0.92) in the biomarker panel, *P*<0.001]. It was suggested that combined with the nodule size significantly improved the performance of the biomarker panel to identify lung cancer patients from normal individuals.

## Discussion

In recent decades, lung cancer has quickly become one of the most predominant and malignant cancer forms in China 1, 26. Though tremendous advances have been made in terms of targeted therapy and immunotherapy for treating lung cancer patients, efficient clinical modules of early detection or prevention, which may bear significant social and economic benefits, are substantially lacking 3.

Previous studies had demonstrated that serum biomarkers of Pro-SFTPB, CA125, Cyfra21‐1, and CEA performed well on an individual basis in the discrimination of lung cancer from healthy controls 11, 22. Particularly, pro-SFTPB showed excellent performance with high AUC values for discerning various subtypes of lung cancers 22, 23. In a recent study, a serum biomarker panel including Pro-SFTPB, CA125, Cyfra21‐1, and CEA was demonstrated to be efficiently predicting the short-term (6~12 months) lung cancer risk in an ever-smoking patient population 11. Thus, the goal of our study was to determine whether this 4-protein serum biomarker panel may have improved performance, as compared with individual biomarker, for detecting lung cancer in Chinese patients. To do so, two models, namely the biomarker panel model and nodule risk model, were independently developed (in the training cohort) and validated (in the validation cohort) for the diagnosis of lung cancer. Our results showed that the performance of the biomarker panel was significantly better than the individual marker in discerning lung cancer patients from a healthy subject. In addition, the sensitivity, specificity, and AUC were almost consistently higher in the nodule risk model than those in the biomarker panel model, in line with an early study showing additional nodule assessment significantly improved the performance of serum protein biomarker panel to identify high-risk lung cancer patients 5.

The most important finding of this study was that we discovered biomarker panel had significantly better AUC than those of individual markers alone for detecting early-stage cancer patients in both training and validation cohorts, thus implicating a clinical potential for lung cancer early diagnosis. Compared with the previous study based on the western population 11, the diagnosis performance of the 4-protein biomarker panel in our study was slightly better (AUC=0.88 in our study vs. AUC=0.80 in the previous study), especially for detecting the early-stage cancer patients (AUC of 0.78 in our study vs. AUC=0.68 in the previous study). It is worth noting that, in our study, early-stage cancer patients represented nearly half of all cases evaluated. Though we do not believe it is the factor to confound the conclusion of this study, future clinical trials with a larger number of participants and heterogeneous sample populations would further refine and develop the 4-protein biomarker panel to be suitable for early lung cancer diagnosis.

In addition to the excellent discrimination for identifying early-stage lung cancer patients, 4-protein biomarker panel was demonstrated to be effectively diagnosing the small malignant pulmonary nodules from normal individuals (AUC=0.89 in the training cohort and AUC=0.90 in the validation cohort). A recent study assessed the performance of the 4-protein biomarker panel in distinguishing benign from malignant pulmonary nodules 27. The performance of the 4-protein biomarker panel for the small nodules (nodule size<1cm) (AUC=0.70) was lower than that in our study (AUC=0.89 in the training cohort and AUC=0.90 in the validation cohort). However, in a small subset (contained 12 cases and 15 controls with nodule size ≤ 6 mm), the 4-protein biomarker panel performed well, with an AUC of 0.95 27. As our sample size of small nodules subgroup (36 cases in the training cohort and 30 cases in the validation cohort) was not large enough, the findings in the current study should be further validated in a larger population.

In comparison to a previous study showing 4 protein biomarkers that can predict short-term lung cancer risk 11, the major difference in our study was that we did not find any individual protein biomarker or prediction model to specifically identify lung cancer patients among current or ever smokers. A major contributing factor, as we noted, maybe that non-smoking lung cancer patients comprised a great proportion (> 60%) in our training and validation sets. However, based on our analysis of individual biomarker and biomarker panel for smoking history, the altered distribution does not appear to account for the difference in performance. Currently, a large‐scale lung cancer screening study, based on a 4-protein biomarker panel model, has been launched and we are expending the enrollment to allow us to more precisely analyze the smoking and non-smoking participants within various lung cancer sub-categories. We expect it to lead us to identify more efficient early diagnosis modules to benefit both smoking and non-smoking lung cancer patients.

## Figures and Tables

**Table 1:** Patient demographics and clinical profiles in the training and validation cohort

Demographics	Training Cohort		*P*-value	Validation Cohort		*P*-value
	Case group	Control group		Case group	Control group	
Overall number	180	360	/	135	289	/
Age, year (mean±SD) (Range)	62.5±10.2 (33-84)	60.7±8.9 (45-88)	0.052	62.2±10.8 (27-83)	58.0±7.7 (27-86)	<0.001
Sex, No. of patients (%)						
Male	111 (61.7)	218 (60.6)	0.803	90 (66.7)	180 (62.3)	0.382
Female	69 (38.3)	142 (39.4)		45 (33.3)	109 (37.7)	
Nodule size, cm (mean±SD)	2.3±2.0^ a^	0.4±0.2^ b^	<0.001	2.6±2.3^c^	0.4±0.2^d^	<0.001
Smoking status, No. of patients (%)						
Never	112 (62.2)	233 (64.7)	<0.001	83 (61.5)	195 (67.5)	<0.001
Current	36 (20.0)	58 (16.1)		34 (25.2)	44 (15.2)	
Former	30 (16.7)	17 (4.7)		18 (13.3)	10 (3.5)	
NA	2 (1.1)	52 (14.4)		0	40 (13.8)	
Stage, No. of patients (%)						
I & II	83 (46.1)	/	/	55 (40.5)	/	/
III & IV	85 (47.2)			69 (51.1)		
NA	12 (6.7)			11 (8.2		
Histological subtype, No. of patients (%)						
SCLC	15 (8.3)			17 (12.6)		
NSCLC	158 (87.8)			112 (83.0)		
Not specified	7 (3.9)			6 (4.4)		

Abbreviations: NA, not available; SCLC, small cell lung cancer; NSCLC, non-small cell lung cancer, including lung adenocarcinoma, lung squamous cell carcinoma, and large cell lung cancer. Statistical analysis was performed using the unpaired two-tail Student t-test (for age and nodule size) and chi-square test (for sex and smoking status). a: Data on nodule size was not available for 55 lung cancer cases. b; Data on nodule size was not available for 272 controls. c: Data on nodule size was not available for 42 lung cancer cases. d: Data on nodule size was not available for 218 controls.

**Table 2:** Serum levels of 4 protein biomarkers in two cohorts with Luminex assay

Attributions		Biomarker levels (mean±SD)			
		Pro-SFTPB (ng/mL)	CA125 (U/mL)	Cyfra21-1 (ng/mL)	CEA (ng/mL)
Training cohort	Control (n=360)	2.6±1.7	2.3±0.6	0.5±0.3	1.7±1.2
	Lung cancer (n=180)	19.3±32.1	9.8±39.5	2.7±11.0	13.5±60.4
	Never smokers (Case set, n=112)	17.5±32.0	10.9±49.1	2.3±10.0	14.7±71.5
	Current smokers (Case set, n=36)	26.5±40.1	7.8±58.6	4.6±17.1	10.9±44.9
	Stage I & II (n=83)	7.9±13.7	3.0±1.3	0.7±0.5	2.4±2.5
	Stage III & IV (n=85)	30.2±40.8	16.4±56.5	3.8±12.8	17.7±50.7
	ADC (n=119)	17.9±34.1	6.4±17.1	0.8±1.1	11.3±42.6
	SCC (n=36)	23.3±26.2	20.2±81.3	7.4±19.1	4.3±5.3
	SCLC (n=15)	9.1±7.4	5.5±3.7	0.7±0.3	10.3±23.4
	Nodule size <1 cm (Case set, n=36)	17.3±31.9	6.9±10.5	1.7±6.1	3.1±3.3
	Nodule size ≥1 cm (Case set, n=89)	16.2±32.1	4.4±4.5	2.0±9.4	3.7±5.6
Validation cohort	Lung cancer (n=135)	16.1±23.0	6.2±9.7	2.4±6.8	12.4±61.4
	Control (n=289)	2.5±1.6	2.4±0.8	0.5±0.4	1.8±0.9
	Never smokers (Case set, n=83)	11.4±17.1	4.9±5.3	2.6±7.5	14.4±76.7
	Current smokers (Case set, n=34)	20.9±23.1	9.5±16.7	2.4±6.7	10.6±25.60
	Stage I & II (n=55)	5.7±5.3	2.9±1.2	0.7±0.4	2.9±5.9
	Stage III & IV (n=69)	22.5±27.1	9.0±12.9	3.5±8.1	20.8±85.1
	ADC (n=78)	13.6±21.7	4.9±7.5	0.8±0.9	17.7±80.4
	SCC (n=31)	16.3±18.6	5.0±5.9	5.7±10.1	3.8±3.6
	SCLC (n=17)	15.1±11.4	10.5±12.0	2.7±8.4	4.0±4.5
	Nodule size <1 cm (Case set, n=30)	17.0±24.3	5.2±4.4	2.3±7.2	4.1±4.5
	Nodule size ≥1 cm (Case set, n=63)	13.8±22.8	4.6±9.0	1.7±5.2	3.0±4.1

Abbreviations: ADC, adenocarcinoma; SCC, squamous cell carcinoma; SCLC, small cell lung cancer; Pro-SFTPB, pro-surfactant protein B; CA125, carbohydrate antigen 125; Cyfra21-1, cytokeratin 19 fragment; CEA, carcinoembryonic antigen.

**Table 3:** Specification of the 4-protein biomarker panel developed based on the training cohort

Variable	Beta Coefficient	OR (95% CI)	*P*
CA125	2.28184	9.7947 (3.2809-29.2405)	<0.0001
CEA	-0.32777	0.7205 (0.3950-1.3143)	0.2852
Cyfra21-1	1.38404	3.9910 (1.8089-8.8052)	0.0006
Pro-SFTPB	1.44898	4.2588 (2.8753-6.3080)	<0.0001
Constant	-3.68815		<0.0001

Abbreviations: 95% CI, 95% confidence interval; OR, odds ratio; CA125, carbohydrate antigen 125; CEA, carcinoembryonic antigen; Cyfra21-1, cytokeratin 19 fragment; Pro-SFTPB, pro-surfactant protein B.

**Table 4:** Sensitivity and specificity of two models in the training cohort and validation cohort

Model		Sensitivity, %	Specificity, %
Biomarker Panel	Training	90.0	61.1
		95.0	47.8
		99.0	27.2
		62.8	90.0
		57.8	95.0
		54.8	99.0
	Validation	90.0	69.3
		95.0	43.9
		99.0	27.8
		69.6	90.0
		60.7	95.0
		47.4	99.0
Nodule risk panel	Training	90.0	92.2
		95.0	75.8
		99.0	44.1
		91.7	90.0
		86.4	95.0
		73.9	99.0
	Validation	90.0	84.8
		95.0	72.7
		99.0	39.2
		85.1	90.0
		82.2	95.0
		70.4	99.0

**Table 5:** The nodule risk model for the probability of lung cancer among participants with nodules in the training cohort

Variable	Beta Coefficient	OR (95% CI)	*P*
CA125	3.59790	36.5216 (1.8645-715.3960)	0.0178
CEA	0.48452	1.6234 (0.2561-10.2903)	0.6071
Cyfra21-1	0.036487	1.0372 (0.2223-4.8379)	0.9630
Pro-SFTPB	1.12225	3.0718 (1.2034-7.8406)	0.0189
Nodule diameter	7.63476	2068.8743 (234.5544-18248.3920)	<0.0001
Constant	-10.10431		<0.0001

Abbreviations: 95% CI, 95% confidence interval; OR, odds ratio; CA125, carbohydrate antigen 125; CEA, carcinoembryonic antigen; Cyfra21-1, cytokeratin 19 fragment; Pro-SFTPB, pro-surfactant protein B.
